# Association of Coronary Wall Shear Stress With Atherosclerotic Plaque Burden, Composition, and Distribution in Patients With Coronary Artery Disease

**DOI:** 10.1161/JAHA.112.002543

**Published:** 2012-08-24

**Authors:** Parham Eshtehardi, Michael C. McDaniel, Jin Suo, Saurabh S. Dhawan, Lucas H. Timmins, José Nilo G. Binongo, Lucas J. Golub, Michel T. Corban, Aloke V. Finn, John N. Oshinski, Arshed A. Quyyumi, Don P. Giddens, Habib Samady

**Affiliations:** 1 From the Division of Cardiology, Department of Medicine, Emory University School of Medicine, Atlanta, GA (P.E., M.C.M., S.S.D., L.H.T., L.J.G., M.T.C., A.V.F., A.A.Q., H.S.); 2 Wallace H. Coulter Department of Biomedical Engineering, Georgia Institute of Technology and Emory University, Atlanta, GA (J.S., L.H.T., J.N.O., D.P.G.); 3 Department of Biostatistics and Bioinformatics, Emory University Rollins School of Public Health, Atlanta, GA (J.N.G.B.)

**Keywords:** atherosclerosis, coronary arteries, computational fluid dynamics, ultrasonography, intravascular, histology, virtual, wall shear stress

## Abstract

**Background:**

Extremes of wall shear stress (WSS) have been associated with plaque progression and transformation, which has raised interest in the clinical assessment of WSS. We hypothesized that calculated coronary WSS is predicted only partially by luminal geometry and that WSS is related to plaque composition.

**Methods and Results:**

Twenty‐seven patients with coronary artery disease underwent virtual histology intravascular ultrasound and Doppler velocity measurement for computational fluid dynamics modeling for WSS calculation in each virtual histology intravascular ultrasound segment (N=3581 segments). We assessed the association of WSS with plaque burden and distribution and with plaque composition. WSS remained relatively constant across the lower 3 quartiles of plaque burden (*P*=0.08) but increased in the highest quartile of plaque burden (*P*<0.001). Segments distal to lesions or within bifurcations were more likely to have low WSS (*P*<0.001). However, the majority of segments distal to lesions (80%) and within bifurcations (89%) did not exhibit low WSS. After adjustment for plaque burden, there was a negative association between WSS and percent necrotic core and calcium. For every 10 dynes/cm^2^ increase in WSS, percent necrotic core decreased by 17% (*P*=0.01), and percent dense calcium decreased by 17% (*P*<0.001). There was no significant association between WSS and percent of fibrous or fibrofatty plaque components (*P*=NS).

**Conclusions:**

In patients with coronary artery disease: (1) Luminal geometry predicts calculated WSS only partially, which suggests that detailed computational techniques must be used to calculate WSS. (2) Low WSS is associated with plaque necrotic core and calcium, independent of plaque burden, which suggests a link between WSS and coronary plaque phenotype. **(*J Am Heart Assoc*. 2012;1:e002543 doi: 10.1161/JAHA.112.002543.)**

## Introduction

Atherosclerosis initiates with fatty streak formation in an atherogenic milieu that is created by the interplay of systemic cardiovascular risk factors, vascular biology, and local hemodynamic forces, including wall shear stress (WSS). WSS is the frictional force of blood exerted tangential to the vessel wall and is dependent on vascular geometry, flow rate, blood properties, and near wall velocity values.^1,2^ Vascular geometric characteristics, such as inner curvatures or outer hips of bifurcations, predispose them to early plaque development^3–6^ that, in turn, changes luminal dimensions, further altering WSS.^7^

Experimental studies have linked low WSS to a variety of atherosclerotic mechanisms and have demonstrated that segments with low WSS develop atherosclerotic lesions with high lipid necrotic core.^8–10^ We have demonstrated a longitudinal relationship between WSS and plaque progression and transformation in patients with coronary artery disease (CAD), establishing an important role for baseline WSS in prediction of atherosclerosis evolution over time.^11^

Because WSS is affected by luminal dimensions (ie, to a first approximation WSS ≍ 1/*r*^3^, where *r* is the lumen radius), some have argued that the local WSS can be estimated from purely anatomic measures such as angiography or intravascular ultrasound (IVUS). However, in addition to luminal geometry, local hemodynamics and ultimately WSS depends on flow rate, blood velocity, vessel angulation, curvatures in multiple planes, and bifurcations. Therefore, we hypothesized that local WSS is predicted only partially by luminal geometry and that WSS is related to plaque composition, providing a link between WSS and vascular pathobiology in human coronary atherosclerosis. Accordingly, we examined the cross‐sectional relationship between local WSS and plaque burden, lesion location, bifurcations, and plaque composition in patients with CAD.

## Methods

### Study Population

Twenty‐seven patients who presented to the cardiac catheterization laboratory at Emory University Hospital between December 2007 and January 2009 with an abnormal noninvasive stress test or stable anginal syndromes and who were found to have nonobstructive lesions requiring invasive physiological evaluation were enrolled. Exclusion criteria included myocardial infarction, cardiogenic shock or hemodynamic instability, a lesion requiring percutaneous or surgical revascularization, severe valvular heart disease, presence of visual coronary collaterals, inability to provide informed consent, serum creatinine >1.5 mg/dL, liver disease, and significant hematologic disease. Eligible patients provided written informed consent. The study was approved by the Emory University Institutional Review Board. A subgroup of these patients underwent repeat evaluation at 6 months to assess the relationship between baseline WSS and plaque progression over time. These data are not included in the present manuscript and were reported elsewhere.^11^

### Coronary Angiography and Physiology Assessment

All patients underwent biplane coronary angiography in a Philips Integris biplane cardiac catheterization system (Philips Medical Systems, Andover, MA). A calibration box with fixed markers in 2 planes was placed beneath the catheterization table, and IVUS imaging was performed in the index vessel to allow 3‐dimensional (3D) reconstruction of the coronary artery. The biplane angles were chosen to visualize the index vessel as well as at least 3 horizontal and 3 vertical markers on the calibration box in each view. At the time of catheterization, a 6‐French coronary guide catheter was introduced into the ostium of the coronary artery. Fifty U/kg of unfractionated heparin was administered intravenously as a bolus. Fractional flow reserve (FFR) and Doppler velocity were measured with a 0.014‐inch pressure and Doppler flow velocity–monitoring guidewire (ComboWire, Volcano Corporation, Rancho Cordova, CA). FFR was performed to assess the hemodynamic significance of the epicardial stenosis for exclusion of patients with hemodynamically significant (obstructive) CAD according to our inclusion/exclusion criteria. The ComboWire was advanced to the tip of the guide catheter, and aortic and guidewire pressures were equalized. The guidewire was advanced into the proximal, nontortuous portion of the vessel at least 5 mm from major angiographic side branches (>2 mm diameter), and the inlet velocity was recorded. The ComboWire was then advanced into the distal vessel and the outlet velocity was also recorded. Subsequently, intravenous adenosine was infused at 140 μg/kg per minute for 3 minutes to induce maximal coronary hyperemia for measurement of FFR. FFR was defined as the ratio of mean distal to aortic pressure during hyperemia.^12,13^

To assess the reproducibility of the resting velocity measurements used in computational fluid dynamics (CFD) models, we performed 2 separate average peak Doppler flow velocity measurements in 3 different locations in each of 10 patients, yielding a total of 60 measurements. We found good reproducibility for average peak velocity (concordance correlation coefficient=0.979 [95% confidence interval (CI) 0.966–0.988]).

### Grayscale and Virtual Histology IVUS Image Acquisition and Analysis

IVUS image acquisition was performed after administration of 200 μg intracoronary nitroglycerin with a phased‐array 20 MHz Eagle Eye Gold Catheter and s5 Imaging System (Volcano Corporation, Rancho Cordova, CA). The IVUS catheter was located as distal as possible by using a fiduciary side branch as the starting point, and an angiographic image was recorded to note the IVUS catheter start position and its relationship to adjacent anatomic landmarks, such as diagonal or septal branches. Automated motorized pullback (0.5 mm/s) was performed, and IVUS images were acquired continuously up to the guide catheter in the aorta to sample 60 mm of the proximal vessel. The ECG‐gated grayscale IVUS images and radiofrequency signals were acquired (peak of the R wave) and stored for offline analysis. Offline volumetric reconstruction and analysis of the entire imaged segment were performed using Volcano Image Analysis Software (VIAS) V3.0 (Volcano Corporation, Rancho Cordova, CA) by a single experienced investigator who was blinded to the patients' clinical and WSS data according to criteria of the American College of Cardiology Clinical Consensus document on IVUS.^14^

IVUS measurements of the external elastic membrane (EEM), lumen, and plaque (plaque and media: EEM – lumen) cross‐sectional areas were performed for every recorded virtual histology IVUS (VH‐IVUS) frame (0.5‐mm thickness, herein defined as an IVUS segment). Plaque burden was calculated as plaque area divided by EEM area.^15^ To assess plaque composition, absolute and relative (percentage) area of VH‐IVUS parameters (fibrofatty tissue, fibrous tissue, necrotic core, and dense calcium) were measured for each IVUS segment.^16,17^ Intraobserver analysis was performed by the experienced analyst in random samples of 10 patients (n=886 frames) at least 2 weeks apart and demonstrated good reproducibility for plaque area (concordance correlation coefficient = 0.968 [95% CI 0.965–0.971]) and necrotic core area (concordance correlation coefficient = 0.978 [95% CI 0.976–0.980]).

Atherosclerotic lesion segments were defined by IVUS segments with plaque burden ≥40% over ≥3 consecutive frames, as defined by the document for standard acquisition, analysis, and reporting of radiofrequency data analysis for IVUS studies.^17^ Distal and proximal segments were defined as segments within 5 mm distal or 5 mm proximal to a lesion.

### Coronary Artery Reconstruction, CFD, and WSS Analysis

Coronary artery reconstruction, CFD modeling, and WSS analysis were performed as previously described.^11^ Briefly, the 3D spatial location of the IVUS catheter during pullback was determined by using the corresponding biplane angiographic projections acquired before pullback. The biplane images were coregistered with a specially designed platform beneath the subject, which contained spatial markers that enabled definition of precise geometric locations in 3D space, independent of any eccentricity of the catheter within the vessel lumen. The 3D‐reconstructed catheter core served as the stem on which to rebuild the geometry. The position of each ECG‐gated IVUS frame was determined from the reconstructed trajectory and speed of catheter pullback. After image adjustment because of rotation, each frame was aligned perpendicular to the catheter core. Subsequent to reconstruction of the main artery of interest, arterial branches were added as cylinders perpendicular to the main artery center line on the basis of information from angiography and IVUS images. This approximation was necessitated by the fact that IVUS visualization of side branches is impractical in the clinical environment. The boundary points of each frame were connected by spline curves to rebuild the luminal geometry. The reconstructed 3D surface was meshed in CFD‐GEOM (CFD Research Corp, Huntsville, AL), and the mesh was imported into the commercial solver CFD‐ACE (CFD Research Corp, Huntsville, AL).

**Figure 1.**
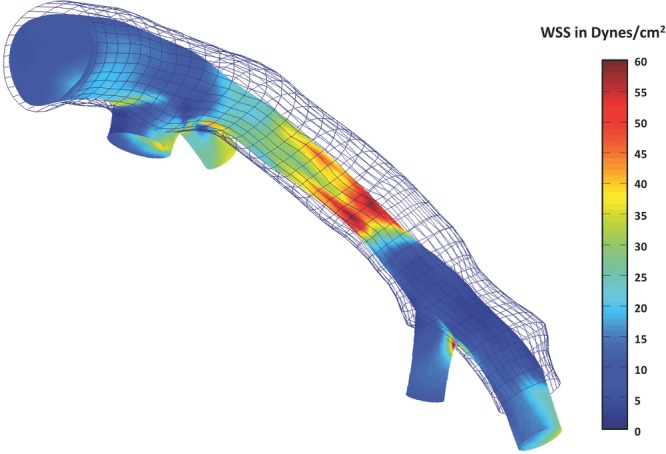
An example of WSS profile of the left anterior descending coronary artery from a study patient, showing areas of variable WSS. Time‐averaged WSS values were circumferentially averaged for each IVUS segment to provide quantitative hemodynamic data to correlate with plaque data. The colors represent different values of WSS as depicted in the scale on the right side. The outer mesh represents the EEM, and the area between EEM and lumen (colored) is considered to be plaque. Each cross‐sectional line in the mesh represents 1 VH‐IVUS frame.

**Figure 2.**
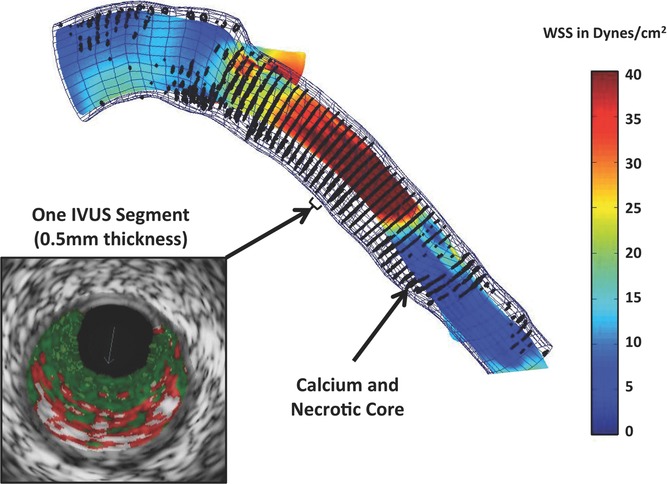
An example of WSS profile of another patient demonstrating heterogeneity of distribution of WSS, which would be difficult to ascertain from geometry alone. The colors represent different values of WSS as depicted in the scale on the right side. Black dots are superimposed VH‐IVUS–derived necrotic core and dense calcium data. A cross‐sectional view of study segment representing 1 VH‐IVUS segment with 0.5‐mm thickness is also shown.

Inlet and outlet boundary conditions were specified as a series of velocity profiles with values from the acquired Doppler data. Our assumption for the inlet velocity was a plug profile with velocity equal to 80% of the peak velocity registered in the Doppler ultrasound sample volume (corresponding to peak values in the Doppler spectrum). This assumption was imposed to account for the fact that the measured Doppler spectra were not sampled over the entire vessel cross section. The profile was imposed at each time step in the pulsatile cycle, as determined from the measurements recorded from a stable set of cycles. The inlet section was extended proximally to provide an entrance length of 1.5 times the vessel diameter to allow for a smooth transition into the computational domain. The fluid (blood) was assumed to be an incompressible Newtonian fluid with a viscosity coefficient of 3.5 centipoise. It is known that the Newtonian assumption for blood is valid under the pulsatile, moderate Reynolds number flow conditions in coronary arteries.^18^ After computing the pulsatile flow field, WSS was determined as a function of time in the cardiac cycle and spatial location around the lumen.^19,20^ Subsequently, the time average of the magnitude of WSS over the pulsatile cycle at each point was computed. Finally, at each IVUS cross section, time‐averaged WSS values were averaged around the circumference. The result is a spatially and temporally averaged value of WSS magnitude for each IVUS cross section. Although WSS is a continuous and dynamic variable, for the purpose of comparison, we used the quartiles of WSS in all 3581 coronary segments. Also, on the basis of previous experimental and human data, low WSS was defined as <10 dynes/cm^2^ and high WSS as ≥25 dynes/cm^2^.^10,11,21,22^ Two examples of WSS profiles derived from CFD calculation are shown in Figures 1 and 2.

### Statistical Analysis

Continuous variables are described as mean and 95% CI (or median and interquartile range [Q1–Q3], as appropriate), and categorical variables as counts and proportions. Logarithmic transformations were performed whenever the distribution of an outcome appeared to be not normal. Repeated‐measures analysis with mixed‐effects linear models was conducted to account for the correlation between multiple observations from the same subject. The model‐based means are unbiased with unbalanced and missing data, so long as the missing at random can be assumed. Valid standard errors were calculated, and efficient statistical tests were performed. A compound symmetric variance‐covariance form among the repeated measurements was used after consideration of other error covariance structures. Concordance correlation coefficient was used to evaluate the reproducibility of the IVUS and velocity measurements.^23^ SAS version 9.2 (SAS Institute Inc, Cary, NC) was used in the data analysis. Statistical tests were 2 sided; the significance level was set at 0.05.

## Results

Twenty‐seven patients were enrolled, yielding 28 left coronary arteries (27 left anterior descending and 1 left circumflex artery) for invasive evaluation. Baseline clinical and demographic characteristics of the study population are summarized in Table 1. All 3581 acquired IVUS segments from the 28 arteries were analyzed, with a median of 128 (Q1–Q3: 100–174) IVUS segments per artery. The median value of WSS in total segments was 23.3 (Q1–Q3: 16.5–33.5) dynes/cm^2^.

**Table 1.** Clinical and Demographic Characteristics of Study Population (N=27 Patients)

Age, y	50 (44–66)
Male, n (%)	16 (60)
White race, n (%)	18 (67)
Body mass index, kg/m^2^	29.8 (26.2–36.2)
Hypertension, n (%)	16 (60)
Systolic blood pressure, mm Hg	128 (116–143)
Diastolic blood pressure, mm Hg	73 (69–85)
Heart rate, bpm	66 (56–80)
Current smoking, n (%)	6 (22)
Diabetes mellitus, n (%)	7 (26)
Hypercholesterolemia, n (%)	7 (26)
Statin use, n (%)	4 (15)
β‐Blocker use, n (%)	11 (41)
Calcium channel blocker use, n (%)	2 (7)
ACE inhibitor or ARB use, n (%)	11 (41)
Family history of CAD, n (%)	12 (44)
Previous myocardial infarction, n (%)	2 (8)
Total cholesterol, mg/dL	181.5 (160.0–204.3)
Triglycerides, mg/dL	114.0 (70.5–152.8)
FFR*	0.93 (0.81–0.96)

Data are number (%) or median (Q1–Q3).

ACE indicates angiotensin‐converting enzyme; ARB, angiotensin receptor blocker.

Hypercholesterolemia was defined as total cholesterol ≥200 mg/dL.*Measured in 28 vessels as described in the Methods.

### WSS, Plaque Characteristics, and Luminal Geometry

**Figure 3.**
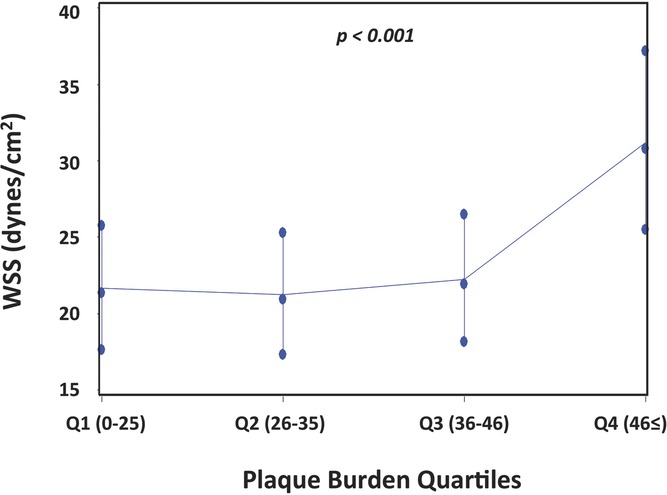
Association between WSS and quartiles of plaque burden. The range of plaque burden in each quartile is shown in brackets. Error bars are 1 standard error.

The grayscale IVUS findings stratified by WSS quartiles are presented in Table 2, which demonstrates that higher WSS was associated with smaller EEM and lumen areas and with greater plaque area and plaque burden (*P*<0.0001 for all comparisons). When WSS and plaque burden were treated as continuous variables and were assumed to have a linear relationship, we observed a positive trend (*P*<0.001). However, the observed positive linear relationship was driven mainly by significantly higher WSS in segments with plaque burden ≥46% (the highest quartile of plaque burden) (Figure 3). In fact, when the analysis was confined to segments with plaque burden <46% (the lower 3 quartiles of plaque burden), no statistically significant association between WSS and plaque burden was observed (*P*=0.08).

**Table 2.** Grayscale IVUS Findings of Study Population Stratified for WSS Quartiles

	WSS 1st Q	WSS 2nd Q	WSS 3rd Q	WSS 4th Q	P*
EEM area, mm^2^	16.3 (14.7–18.0)	15.3 (13.8–16.9)	14.8 (13.4–16.4)	14.7 (13.3–16.2)	<0.0001
Lumen area, mm^2^	10.7 (9.6–12.0)	9.8 (8.8–11.0)	8.9 (8.0–9.9)	7.5 (6.7–8.4)	<0.0001
Plaque area, mm^2^	5.1 (4.2–6.2)	4.8 (4.0–5.8)	5.2 (4.3–6.3)	5.8 (4.8–7.0)	<0.0001
Plaque burden, %	31.1 (27.0–35.8)	31.4 (27.3–36.1)	34.8 (30.3–40.0)	39.4 (34.3–45.3)	<0.0001

Q indicates quartile. Model‐based means for each category are reported as mean (95% CI).

* Mixed‐effects models were used to account for repeated measurements.

**Figure 4.**
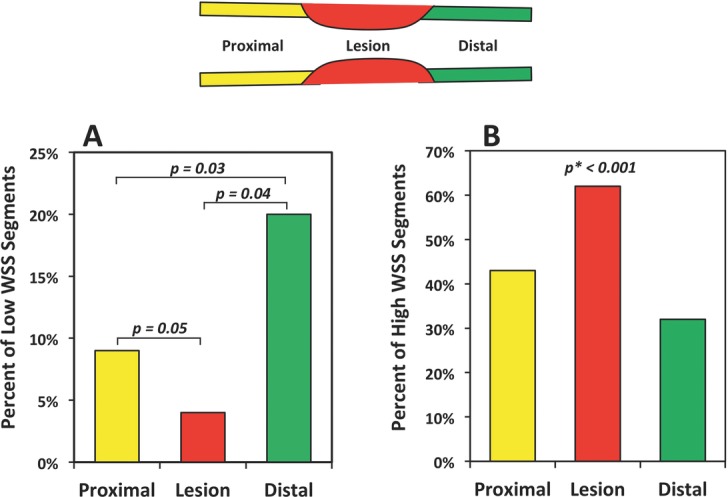
A, Percentage of segments with low WSS (<10 dynes/cm^2^) within the lesions and proximal to and distal to lesions. B, Percentage of segments with high WSS (≥25 dynes/cm^2^) within the lesions and proximal to and distal to lesions. *P value: The GLIMMIX procedure in SAS did not converge when fitting the statistical model for Figure 4B. Convergence was achieved when lesion and distal were consolidated into 1 category.

We then investigated the relationship between WSS and lesion location and bifurcations. Of the total 3581 IVUS segments, 1390 resided within a lesion, 226 were proximal to a lesion, 252 were distal to a lesion, and 1713 were neither within lesions nor proximal or distal to lesions. Mean WSS was significantly higher within lesions (26.6 [95% CI 21.1–33.6]) dynes/cm^2^) than proximal (24.3 [95% CI 19.2–30.8] dynes/cm^2^) or distal to lesions (18.9 [95% CI 14.9–28.9] dynes/cm^2^; *P*<0.0001). Interestingly, WSS was lower in distal segments than in the proximal segments (*P*=0.02), despite the similar amount of plaque burden (31.4 [95% CI 30.6–32.3] versus 30.5 [95% CI 29.7–31.4]; *P*=0.79) and smaller lumen (9.1 [95% CI 8.7–9.5] versus 11.8 [95% CI 11.2–12.5] mm^2^; *P*<0.0001). In addition, segments distal to lesions were more likely to have low WSS (<10 dynes/cm^2^) than were segments within lesions (20% versus 4%; *P*=0.04) or proximal to lesions (20% versus 9%; *P*=0.03) (Figure 4A). Conversely, segments within lesions were more likely to have high WSS (≥25 dynes/cm^2^) than were segments residing proximal or distal to lesions (62% versus 43% and 32%; *P*<0.001) (Figure 4B). The corollary to the relationship between WSS and lesion location is that 80% of segments distal to lesions did not have low WSS and 38% of segments within lesions did not have high WSS.

**Figure 5.**
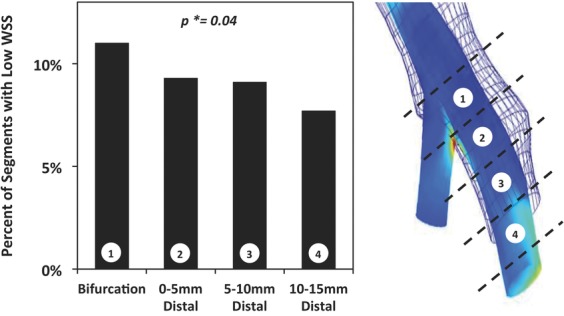
Percentage of segments with low WSS (<10 dynes/cm^2^) within the bifurcations, and 0 to 5 mm, 5 to 10 mm, and 10 to 15 mm distal to bifurcations.

The distribution of segments with low and high WSS in relation to bifurcations is shown in Figure 5. Although the percentage of segments with low WSS decreased from within the bifurcation to distal to the bifurcation, with the highest percentage within the bifurcation and the lowest percentage within 10 to 15 mm distal to bifurcations (from bifurcation to distal: 11.3% versus 9.3% versus 9.1% versus 7.7%; *P*=0.04), the majority of segments within bifurcations (88.7%) did not exhibit low WSS.

### WSS and Plaque Composition

To account for the expected relationship between plaque burden and plaque components, we assessed the relationship between WSS and 4 plaque components after adjusting for plaque burden. We observed a negative association between WSS and percent necrotic core and percent dense calcium. For each 10‐dynes/cm^2^ decrease in WSS, percent necrotic core increased by 17% (*P*=0.01), and percent dense calcium increased by 17% (*P*<0.001). There was no significant association between WSS and percent fibrous (*P*=0.12) and fibrofatty tissue (*P*=0.58).

## Discussion

When data for all segments measured in study patients were analyzed, we found that: (1) WSS remained relatively constant across the lower 3 quartiles of plaque burden (0% to 46%) and increased in segments within the highest quartile of plaque burden (≥46%). (2) Although segments immediately distal to lesions and within bifurcations were more likely to have low WSS than those not in these locations, the *majority* of segments distal to lesions (80%) and within bifurcations (89%) did not exhibit low WSS. (3) An inverse relationship exists between WSS and percent necrotic core and dense calcium after adjustment for plaque burden. These data demonstrate that luminal geometry predicts calculated local WSS only partially and imply that use of geometry alone for classifying WSS is unreliable. Furthermore, our data suggest that WSS is related to plaque composition independently of plaque burden, providing evidence for a link between WSS and atherosclerotic plaque phenotype in human coronary atherosclerosis.

Extensive experimental investigations in cell culture, animal models, and human subjects support the concept of an important role for WSS in the initiation and progression of atherosclerosis.^8–10,24–26^ Several human studies have now demonstrated a longitudinal relationship between low coronary WSS and plaque progression.^11,27,28^ In addition, we have observed previously that in patients with CAD, coronary segments with high WSS exhibit plaque transformation over time despite high‐dose statin therapy.^11^ Taken together, these data provide a rationale for the potential evaluation of WSS in patients with nonobstructive CAD.

Given the complexity of coronary artery geometry and flow conditions, it is not surprising that reliance on simplified fluid mechanics principles (eg, Poiseuille flow) might not accurately estimate WSS values. Lumen area variations, vessel curvature, flow splits among branches, and the state of distal vessels, including the microcirculation, all contribute to the flow field in a region of interest. Fundamentally, WSS is a highly localized quantity that varies over 3 spatial dimensions and time, and hence determination of WSS solely from visual interpretation of angiographic images can be misleading. In our study population, we found that although segments immediately distal to lesions were more likely to have low WSS, only 20% of these segments actually displayed low WSS, with 80% of segments distal to lesions not demonstrating low WSS. In addition, only 64% of segments within lesions demonstrated high WSS, and we found no statistically significant relationship between WSS and plaque burden until the plaque burden reached 46%. These findings undoubtedly result from the local flow and geometric conditions, because plaque burden does not alter WSS directly until it causes lumen encroachment. Furthermore, if a lesion causes localized encroachment (stenosis, with elevated WSS), the distal distance required to recover WSS values that are comparable to those found proximally is extended.^29^

Similarly, although the proportion of segments with low WSS was greater within bifurcations than distal to bifurcations, the majority of segments within bifurcations did not exhibit low WSS, as one would anticipate from lower WSS values expected at the hips of bifurcations.^30,31^ One potential explanation for this observation could be underestimation of WSS because of circumferential averaging of high as well as low WSS in a given cross section. Taken together, these data suggest that low WSS segments cannot be determined solely by anatomic evaluation and require state‐of‐the‐art computational modeling techniques, such as those deployed in the present study.

Another important finding of this study is that there appears to be an association between WSS and pathobiology. Indeed, after adjustment for plaque burden, segments with lower WSS were associated with higher percent necrotic core and dense calcium, in keeping with experimental data and suggesting an association of low WSS with a more vulnerable plaque phenotype.^8–10,32,33^ To the best of our knowledge, this is the first description of a relationship between low WSS and coronary plaque with high‐risk features in patients with CAD. The observation that low WSS is associated with increased necrotic core and calcium when data are adjusted for plaque burden suggests that the proatherogenic effects of low WSS might be manifest beyond plaque initiation and early disease. Our data are, however, consistent with the concept that not only do plaques originate in areas of low WSS but that the WSS remains low as plaques progress, until the plaque burden causes encroachment of the lumen, after which WSS increases because of the local narrowing.^2,8,34,35^ All these phenomena can occur before disease progression to the point of flow restriction.

A significant body of experimental literature supports this clinical observation. Low WSS results in alterations in endothelial cell signaling, low‐density lipoprotein uptake, and proinflammatory gene expression, all of which lead to an activated endothelial phenotype.^2,10,34,36,37^ Subsequent in vivo experimental animal models also supported the atherogenic role of low WSS.^9,38^ In a study performed in diabetic hyperlipidemic swine, segments with the lowest WSS at baseline developed larger lipid plaques with inflammation and fibroatheromas at follow‐up.^9^ A recent study has demonstrated that regions of preceding low WSS have reduced endothelial coverage, augmented infiltration of activated inflammatory cells, and substantially increased expression and enzymatic activity of extracellular matrix–degrading enzymes, ultimately promoting the formation of atheromas with thin fibrous cap.^10^ Detailed CFD simulations and in vitro experimental studies in autopsy‐based models of coronary arteries,^39^ carotid bifurcations,^1,7,40–42^ and distal abdominal aortas^43^ also demonstrated an association of low WSS with the localization of atherosclerosis.

Only 2 previous studies have described the relationship between WSS and plaque thickness in human coronary arteries. The first was a study from a single human right coronary artery, where WSS calculations were based on 3 predefined velocity values and no velocity measurements were performed.^44^ The second study demonstrated a positive relationship between circumferentially averaged WSS and wall thickness in 12 angiographically normal coronary arteries (area stenosis <10% by IVUS). Despite its small size, minimal CAD, and relatively low average WSS (14.1±3.2 dynes/cm^2^), the findings of this study support our results.^45^

The present study is unique for several reasons. First, it is the largest in vivo evaluation of the cross‐sectional relationship between local WSS, plaque burden, and VH‐IVUS–derived plaque composition in patients with CAD. As such, it establishes a basis for relationships of the spectrum of WSS with plaque burden, composition, and distribution with regard to lesion location and bifurcations in human coronary arteries. Second, the study population represents a significant spectrum of plaque burden (range: 1% to 64%), which enables us to demonstrate the relationship of WSS with different levels of atherosclerotic plaque. Of note, although patients had functionally nonobstructive CAD (median FFR 0.93 [Q1–Q3 0.81–0.96]), in some cases their coronary arteries had sufficient plaque encroachment to result in blood flow acceleration, creating high values of WSS. Third, our observation that WSS is lower distal to lesions offers, for the first time, some insights as to a potential role of lower WSS in distal coronary plaque progression. Fourth, we present a comprehensive methodology for in vivo assessment of WSS from 3D‐reconstructed geometries that include bifurcations and pulsatile inlet boundary conditions derived from Doppler velocity measurements. Finally, by superimposing local plaque composition from VH‐IVUS with corresponding WSS, this investigation allows unique insights into the relationship between the atherosclerotic content and hemodynamic milieu. Such combination profiling could provide incremental value for identification of plaques that develop rapid progression or transformation to a phenotype at high risk for future acute coronary events that could potentially be approached by intensive local or systemic therapies to reduce future adverse events.

### Limitations

Several limitations should be considered when the results of the present study are interpreted. Although the number of patients in this study is limited, it is the largest study of WSS calculation in humans with CAD, and appropriate statistical methods were used to adjust for correlated error introduced by the clustering of numerous arterial segments within patients. Second, plaque composition data are derived from VH‐IVUS. Although it is the “gold standard” for assessment of plaque composition is true histology, VH‐IVUS cannot be performed in coronary arteries in vivo. Nevertheless, several studies have evaluated the accuracy of VH‐IVUS with predictive accuracies of 87% to 97%,^16,46,47^ and a recent large clinical trial has linked findings of VH‐IVUS in conjunction with grayscale IVUS to adverse clinical outcomes.^48^ Third, although the study population represents a spectrum of atherosclerosis, from mild to moderate disease, flow‐limiting lesions were excluded in the present study. Therefore, our observations are confined to lesions that are not flow limiting. Fourth, WSS magnitudes were averaged temporally and spatially (around the vessel circumference) for each IVUS cross section and do not allow for a highly localized examination of the relationship between WSS and plaque composition. Finally, the present study does not demonstrate any cause–effect relationship between altered WSS patterns, plaque development, and vulnerability process. Prospective studies in patients with CAD are under way to further elucidate the cause–effect relationships of WSS and atherosclerosis.

### Conclusions

In patients with CAD: (1) Luminal geometry predicts calculated WSS only partially, which suggests that detailed computational algorithms must be used to calculate WSS. (2) Low WSS is associated with plaque necrotic core and calcium independent of plaque burden, which suggests a link between WSS and coronary plaque phenotype.
